# Ayurvedacharya K. Kunjuraman Vaidyar: A Kerala-based Physician-Academician and Gandhian Apostle, synthesizing Ayurveda with modern clinical insights

**DOI:** 10.1016/j.jaim.2026.101403

**Published:** 2026-10-14

**Authors:** C.K. Saniya, G.N. Sree Deepthi, T.P. Sinimol, A. Abhayadev

**Affiliations:** a CCRAS-Regional Ayurveda Research Institute, Thiruvananthapuram, Kerala, India; b CCRAS-Central Ayurveda Research Institute, Kolkata, West Bengal, India

## 1 A portrait of Ayurvedacharya K. Kunjuraman Vaidyar

Ayurveda has evolved through many scholars over time, with Kerala standing out in its refinement and growth. The *Ashtavaidyas* and traditional physician families from Kerala made remarkable contributions to its growth in the early days [1].

Among those who left an enduring legacy, Ayurvedacharya K. Kunjuraman Vaidyar (KKV) stands out as a remarkable figure (Fig. 1). Although his life unfolded during the Kerala Renaissance, he came of age at a time when rigid caste hierarchies and upper-class dominance over land and education, particularly Sanskrit learning, remained deeply entrenched. Despite these formidable barriers, he persevered to master Sanskrit and complete advanced studies in Ayurveda. He was far more than a physician; he was an academic and a social activist committed to the progress of the marginalized.

**Fig-1:**
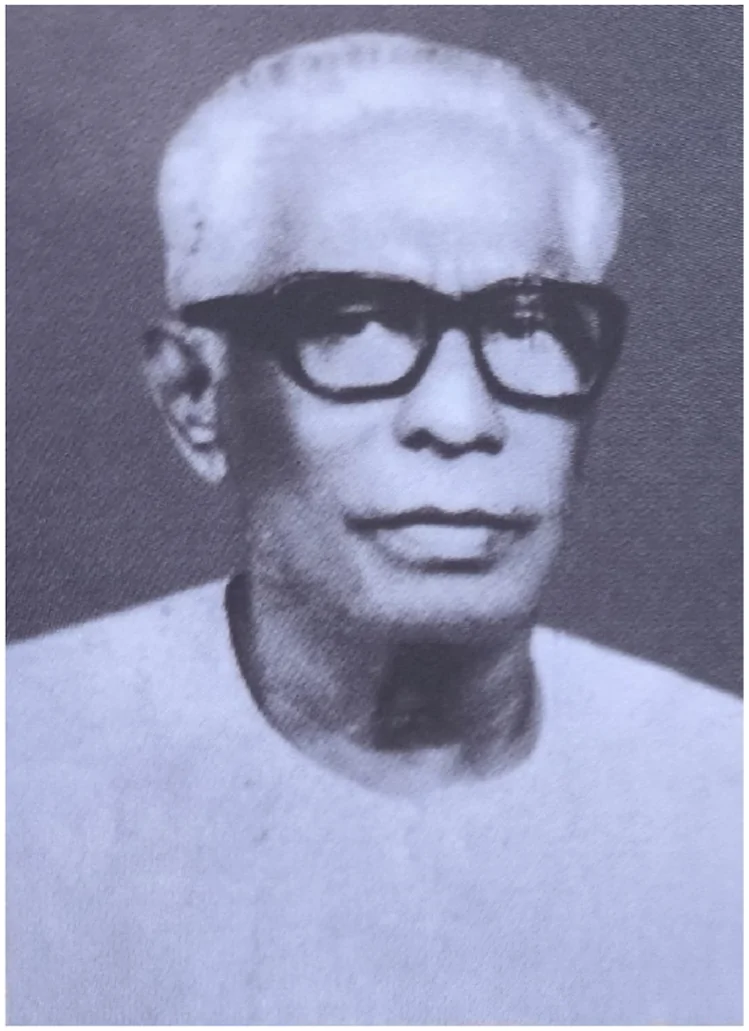
K. Kunjuraman Vaidyar (1918-1993).

By deciding to base his practice in his hometown of Perinadu, Kollam and founding "*Paurastya Pharmacy*," KKV played a crucial role in the area's socioeconomic and public health developments. His efforts during this period are historically highly significant, demonstrating his dedication to the development of Ayurveda and social development.

## 2 Conversations and chronicles: Tracing a Luminary's journey

We conducted an extensive one-day interview using a structured, open-ended framework developed by the Central Council for Research in Ayurvedic Sciences (CCRAS) for the Ayurveda Physicians to Transform Ayurveda (APTA) project. This session involved family members and close associates of KKV, with his son, Dr K. Jyothilal, providing the primary personal insights.

We compiled the gathered information into a narrative manuscript, ensuring the family reviewed and consented to the final version. To maintain historical accuracy, we cross-verified personal details with accessible publications and documents. Any gaps identified during the writing process were clarified through follow-up telephonic interviews. The accompanying photograph is from the personal collection of Dr K. Jyothilal.

Our documentary research included a commemorative souvenir from the centenary celebration of Government Ayurveda College, Thiruvananthapuram. Furthermore, the third edition of ‘*Adhunika Rogyabhaishajyam’* provided vital context, particularly through the preface by Tirunellur Karunakaran, which highlights his significant professional and social contributions.

## 3 Early life and education: shaping a vision for healing

KKV was born in 1918 at Astamudi, Trikkaruva, in the Kollam district of Kerala, a place surrounded by lakes and backwaters. He was the elder of two sons born to Krishnan Vaidyan and Kalyani Amma. His early intellectual orientation was deeply influenced by his father, Krishnan Vaidyan, a traditional Ayurveda practitioner whose work served as a vital healthcare lifeline for a community geographically isolated by backwaters. Growing up in an environment where Ayurveda was not just a profession but a social necessity, the young Kunjuraman developed a foundational understanding of medicine as a tool for community service.

His formal education began at Kavuvila Sanskrit School, followed by the successful completion of the *Sanskrit Sastri* examination at the age of fourteen. This strong linguistic and classical foundation paved the way for his enrolment (circa 1932–1935) in the *Vaidya Kalanidhi* -the degree course started in 1917 at the Government Ayurveda College, Thiruvananthapuram. Rather than merely a period of academic study, this time was marked by a deepening commitment to the science of healing.

A defining moment in his professional formation occurred during his subsequent clinical training under the eminent physician Shri Kumarakam Parameshwaran Pillai. This mentorship provided with exposure to high-level clinical expertise; his teacher notably served as one of the Chief Physicians to the Prime Minister of India and later became the Principal of the Government Ayurveda College. This period was a pivotal turning point, exposing him to leadership and excellence that would later define his own career.

His thirst for knowledge subsequently led him to the *Ayurveda Acharya* (research degree course introduced in 1929 at Government Ayurveda college, Thiruvananthapuram), an advanced pursuit that solidified his scholarly authority and earned him the *Shrimulam Medal*. Although his academic record led to a faculty appointment at the college at the young age of 21, his intellectual and social commitments drew him back to his roots. After eighteen months of teaching, he chose to resign and return to the underserved region of Perinadu. There, he established ‘*Paurastya Pharmacy’* in Cittayam, translating his academic training into a grassroots clinical practice dedicated to the coir workers and lower socio-economic communities of Kollam.

## 4 Family, intellectual lineage and the continuity of tradition

Following his formal education, Kunjuraman Vaidyar married Bhavani, whose supportive presence provided the foundation for a household dedicated to both service and scholarship. Their residence, ‘*Sevasadanam’* in Cittayam, became a centre for the transmission of Ayurvedic knowledge across generations. The couple was blessed with nine children: five sons and four daughters.

The most significant aspect of Kunjuraman Vaidyar's personal life was his success in institutionalizing his family's traditional heritage through formal modern education. All five of his sons built careers within the Ayurvedic field, effectively bridging the gap between ancestral wisdom and contemporary medical education. This transition is best represented by his eldest son, Dr. P.K. Mohanlal, who rose to become the first Director of Ayurveda Medical Education (DAME) in Kerala. Similarly, his second son, Dr. K. Jyothilal, served as a Professor at the Government Ayurveda College, Thiruvananthapuram.

The family legacy continues through his other sons-Pyarilal, who manages the manufacturing at *Paurastya Pharmacy*, and Dr. Jeevanlal and Dr. Aniyanlal, both noted practitioners. By ensuring that his descendants received high-level academic training while remaining rooted in the family's *Vaidya* heritage, KKV secured a remarkable continuity of medical tradition that extends into the third generation of his family.

## 5 Professional life

### 5.1 Teaching legacy and institutional contribution

KKV's professional formation was shaped by institutional scholarship and clinical mentorship. Throughout his life, he remained an intellectual pillar for the academic community, serving as an honorary consultant in the postgraduate division of his alma mater and contributing to the Kerala Government Ayurveda *Upadesaka Samiti*. His commitment to education extended far beyond official titles. His ‘*Paurastya Pharmacy’* became a sanctuary for learning, where students who had completed their formal Ayurvedic degrees would seek him out for practical guidance. Without hesitation, he transformed his clinic into a living classroom, generously sharing his clinical wisdom and teaching the nuances of the science to any aspiring physician.

His dedication to mentorship, along with leadership roles such as President of the ‘Kollam District Ayurveda Cooperative Society’ and membership in the *‘Vaidya Maha Sabha’*, reflects a sustained commitment to the professional organization of Ayurveda practitioners. By serving as Treasurer of the ‘*Ayurveda Mandalam*’ and Principal Physician in the cooperative hospital, he contributed to strengthening institutional and cooperative structures that supported the growth of Ayurveda in Kerala. Collectively, these roles illustrate how his leadership extended beyond clinical practice to the broader consolidation and community-oriented development of the profession.

In the field of Ayurveda, he maintained close associations with several distinguished contemporaries, including Vaidyabhuṣaṇam Raghavan Thirumulppadu, Dr. K. Rajagopalan, Madhavan Vaidyar of Kochi, and Poruttiyil Narayaṇan Vaidyar. These associations highlight his participation in the collaborative professional networks that shaped Ayurvedic practice in Kerala.

### 5.2 Literary and scholarly engagement: bridging science and society

He maintained a sustained engagement with literary activity, dedicating much of his leisure time to reading and writing. He authored poems in Malayalam during the period of the independence movement and in relation to the concerns of coir labourers, and also composed songs for dance performances. His writings in newspapers and periodicals addressed themes related to Ayurveda and public health, often presented in accessible language for a wider readership. One such example is his article titled *‘Janapadoddhvamsaniyam’* (Epidemiological concept narrated in Charaka samhitha), published in *Matṛbhumi Weekly*, where he discussed environmental disturbances and their implications in the light of Ayurvedic concepts.

Through such contributions, his literary engagement functioned as a medium for communicating Ayurvedic ideas within broader cultural and public discourses.

His interactions with contemporary Malayalam literary figures, including O.N.V. Kurup, Tirunellur Karunakaran, and Kambissery Karunakaran, further situate his work within the intellectual and cultural networks of the period.

### 5.3 Gandhian philosophy in practice and social commitment

KKV's professional life also intersected with the social reform movements of his time. Although he was associated with communist ideological currents, he remained influenced by Gandhian principles of non-violent social transformation. A notable episode in his life was his participation in the Temple Entry Proclamation movement of 1936 in Thiruvananthapuram. As part of the symbolic act associated with this reform, he was one among four individuals escorted into the *Padmanabhaswamy Temple*, marking a moment connected with the broader challenge to caste-based exclusion. His admiration for Mahatma Gandhi was also reflected in his personal life, where his children addressed him as “*Bappu*,” rather than the customary Malayalam term “*Achan*” for father and his residence was named as ‘*Sevasadanam*’.

Such social commitments were also reflected in his professional work. His association with institutions such as *Paurastya Pharmacy* and the later establishment of ‘*Bhavani Arogya Nikerthanam’* in memory of his wife indicate an orientation toward community-oriented healthcare. His literary contributions addressing the conditions of coir labourers and his medical service in the backwater regions suggest the intersection between clinical practice with broader social concerns.

## 6 Clinical ethos and scholarly authority

KKV's practice was defined by a clinical ethos that integrated medical proficiency with a commitment to socioeconomic equity. He prioritized healthcare access for disadvantaged communities, effectively operationalizing the Ayurvedic principle within a practical, social framework. This approach shifted the focus of his practice from mere theoretical application to a socially responsive medical model.

His contribution was further characterized by an advanced mastery of classical Sanskrit literature, which served as a primary pedagogical tool. This scholarly authority is evidenced by an interaction recorded by the poet Tirunellur Karunakaran during the translation of Dr. Devi Prasad's *Defence of Action in Indian Medicine*. When consulted on the specific dissection procedures outlined in the *Sushruta Samhita*, KKV provided the precise technical verses from memory. This capacity for verbatim recall and immediate elucidation of complex texts highlights his role as a living repository of the *Vaidya* tradition, providing a bridge between ancient textual protocols and contemporary scholarly inquiry.

## 7 Literary contributions towards Ayurveda

Besides being a distinguished physician, KKV was also an accomplished writer. His literary brilliance was evidenced by the highly popular column ‘*Sundarikal Sumangalikaḷ’*, which he authored for ‘*Janayugaṃ’* weekly in Malayalam. This series, which explored Ayurvedic concepts for beauty, health, and wellness in an engaging and accessible format, was subsequently combined into a book of the same title.

His contributions extended beyond journals, as he produced numerous academic works, predominantly on Ayurveda. Some of these texts also functioned as well-reasoned replies to allopathic physicians who criticized Ayurveda. His essays exhibited logical clarity and linguistic elegance. His second significant literary contribution is *‘Adhunika Rogyabhaishajyam****’***, a thorough and authoritative text on Ayurvedic treatments.

### 7.1 ‘Adhunika Rogyabhaishajyam’ [2]

The book's first edition was published in 1978 and released by then Health Minister Sri J. Chittaranjan, followed by a second edition in 1983; both were printed by Sree Narayana Press and published by Sevasadanam Trust. The third edition was brought out in 2003 by the Ayur-Rekha Foundation, while the fourth edition was planned in collaboration with the Kerala Bhaṣa Institute. An English translation, prepared by his eldest son, Dr. P. K. Mohanlal, is set to be published by Chowkhamba Publishers. The book was recognized as a reference text and was awarded a grant from the Kerala Sahitya Academy.

Kumarakom Parameshwaran Pillai, who wrote the foreword for *‘Adhunika Rogyabhaishajyam*, aptly described its significance. The book systematically examines modern diseases through the lens of contemporary medical science, analyzing their symptoms, variations, and clinical presentations while integrating them with the Ayurvedic framework of *Tridoṣa* (three fundamental bodily humors in Ayurveda: *Vata, Pitta,* and *Kapha*), *Saptha dhathu* (seven body tissues) and *Trimala* (Three main excretory wastes of human body) principles. It elucidates disease pathology and provides insights into Ayurvedic treatment approaches from his experiences rooted in popular Ayurvedic classical texts. By bridging modern medical knowledge with traditional Ayurvedic diagnostics, the book serves as a valuable resource for practitioners seeking to apply Ayurvedic principles in a structured and scientific manner.

The extensive footnotes compiled by Ayurvedācārya K. Kunjuraman Vaidyar in *Adhunika Rogyabhaishajyam* draw upon the *Brihatrayee,* the *Laghutrayee*, other classical Ayurvedic texts, and rare Malayalam medical manuscripts, thereby enhancing the scholarly value of the work through comprehensive cross-referencing and contextual interpretation. By consolidating these diverse sources, the book offers practitioners a "single-glance" access to clinically verified *Yogas* (formulations). In an era of digital information overload, the book's true relevance lies in this curated, expert-validated collection of medicinal descriptions, preserving knowledge that is often inaccessible in modern databases.

Modern medical science has made tremendous strides by leveraging technological advancements and research findings. While Ayurveda does not necessitate the development of complex diagnostic instruments, it can certainly benefit from the knowledge generated through modern scientific methods. The true potential of Ayurveda lies in understanding the world through contemporary scientific advancements while remaining rooted in its therapeutic principles. ‘*Adhunika Rogyabhaishajyam’* is a testament to this philosophy, providing a refined and systematic approach to integrating modern medical insights with Ayurvedic wisdom.

## 8 Conclusion

Every region has its heroes who shaped the evolution of Ayurveda, and KKV was one such figure from south Kerala. This biography revives the memory of a forgotten pioneer of Ayurveda from Kerala. His six-decade-long journey as a clinician, author, social activist, and scientific thinker left a lasting impact on Ayurveda. Committed to healing and teaching, he dedicated himself to Ayurveda.

## Author contributions

**SCK**: Conceptualization, literature review, data collection, drafting, and editing; **SGN**: Data collection, and editing; **STP:** Review and editing;**AA:** Review and editing.

## Declaration of generative AI in scientific writing

During the preparation of this work, the authors used ChatGPT in order to improve the readability and language of the manuscript. After using this tool/service, the authors reviewed and edited the content as needed and takes full responsibility for the content of the publication.

## Funding source

Central Council for Research in Ayurvedic Sciences, Ministry of AYUSH, New Delhi.

## Declaration of competing interest

None.
